# The Curious Case of Nonrepetitive Centromeric DNA Sequences in Candida auris and Related Species

**DOI:** 10.1128/mBio.01476-21

**Published:** 2021-08-03

**Authors:** Alexander Lorenz, Nicolas Papon

**Affiliations:** a Institute of Medical Sciences (IMS), University of Aberdeen, Aberdeen, United Kingdom; b Univ Angers, Univ Brest, GEIHP, SFR ICAT, Angers, France

**Keywords:** *Candida auris*, centromeres, chromosomes, karyotype evolution, centromere

## Abstract

2009 saw the first description of Candida auris, a yeast pathogen of humans. C. auris has since grown into a global problem in intensive care settings, where it causes systemic infections in patients with underlying health issues. Recent whole-genome sequencing has discerned five C. auris clades with distinct phenotypic features which display genomic divergence on a DNA sequence and a chromosome structure level. In the absence of sexual reproduction in C. auris, the mechanism(s) behind the rapid genomic evolution of this emerging killer yeast has remained obscure. Yet, one important bit of information about chromosome organization was missing, the identification of the centromeres. In a recent study, Sanyal and coworkers (A. Narayanan, R. N. Vadnala, P. Ganguly, P. Selvakumar, et al., mBio 12:e00905-21, 2021, https://doi.org/10.1128/mBio.00905-21) filled this knowledge gap by mapping the centromeres in C. auris and its close relatives. This represents a major advance in the chromosome biology of the *Candida*/*Clavispora* clade.

## COMMENTARY

**FIG 1:**
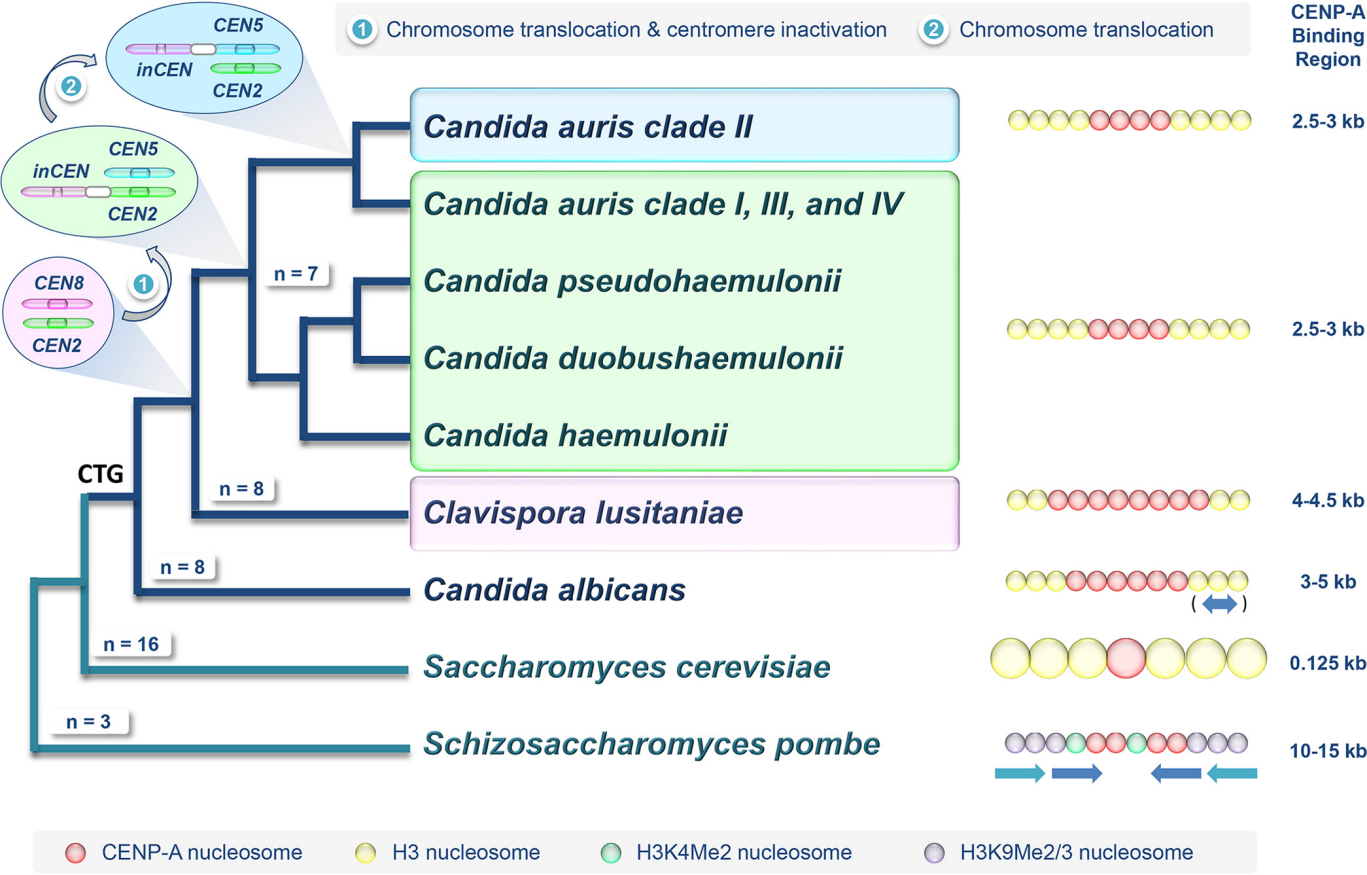
Tracing the evolution of centromeres in C. auris and other ascomycetous species. Branch lengths of the phylogenetic tree are arbitrary, but topologies resemble the current understanding of the relationships of the Metschnikowiaceae (including C. auris, *C. haemulonii*, *C. pseudohaemulonii*, and *C. duobushaemulonii*, and C. lusitaniae) (16). The model yeasts Saccharomyces cerevisiae and Schizosaccharomyces pombe, as well as C. albicans, are also indicated. A first event of chromosome translocation and centromere inactivation in an ancestor of Metschnikowiaceae likely resulted in chromosome number reduction in species belonging to the *C. haemulonii* complex (including *C. haemulonii*, *C. pseudohaemulonii*, *C. duobushaemulonii*, and C. auris). Another chromosome translocation further repositions the inactive centromere in C. auris clade II. Centromeres are numbered using C. lusitaniae as the reference. On the right side a schematic representation of centromere types (not to scale) and lengths of the CENP-A-positive core centromeres for each species/clade are given. Arrows for C. albicans and Schizosaccharomyces pombe indicate DNA repeats in pericentromeres; in the former, repeats do not occur in every pericentromere.

Centromeres are essential features of chromosomes; they are sites where kinetochores are assembled which connect the chromosomes to the microtubular spindle to segregate them during mitosis and meiosis (1). Considering their conserved and fundamental role, a surprising diversity of centromere sequences and types is present in eukaryotes. In fungi, different centromere categories have been described, ranging from the tiny sequence-specific point centromeres of *Saccharomyces*, which represent the pinnacle of genome streamlining, to the large epigenetically defined regional centromeres flanked by repetitive DNA in *Schizosaccharomyces* (Fig. 1) (2). Common to centromeres in most organisms is a special histone H3 variant called CENP-A (centromeric protein A, usually called Cse4 in fungi), which replaces histone H3 in centromeric nucleosomes, where it forms the recruiting platform for kinetochores (1, 2). Thus, in recent studies centromeric DNA in fungi was identified through its propensity to be bound by CENP-A and other kinetochore components (3–7). Fungal centromeric DNAs are often found in large regions of low GC content, so-called GC-poor troughs (8). A notable exception to the latter is Candida albicans, which harbors small regional centromeres (∼3 to 5 kb) defined by unique nonrepetitive sequences (Fig. 1) (3, 8). In C. albicans, the pericentromeric regions can contain repetitive DNA and have features reminiscent of heterochromatin as gene expression is repressed in the vicinity of centromeres and some fitting histone modifications are present (3, 9). *Clavispora* (*Candida*) *lusitaniae* has somewhat similarly sized (∼4.0 to 4.5 kb) centromeres with unique sequences (Fig. 1), but these are located in GC-poor troughs, lack typical heterochromatin marks, and do not suppress gene expression in their vicinity (4).

Candida auris, Candida haemulonii, Candida pseudohaemulonii, and Candida duobushaemulonii make up the Candida haemulonii complex which, together with C. lusitaniae, belongs to the *Clavispora* clade of the Metschnikowiaceae (10). This group of *Clavispora* yeasts is part of the *Candida* CTG clade which also contains C. albicans and its close relatives (11). Since its first identification in 2009 (12), C. auris has become known as a nosocomial yeast pathogen (13). The species has been subdivided into 5 geographical clades; the genomes of the different clades show substantial differences between them on a DNA sequence and a chromosome structure level (14–18). The karyotype (chromosome complement) of C. auris is thus fairly plastic and can change quickly upon stress exposure (17). This karyotype plasticity could at least in part explain how C. auris generates genetic diversity in the absence of sexual reproduction and meiosis (19). What role centromeres play in the karyotype diversification of C. auris and other *Clavispora* yeasts could not be answered until recently, when Sanyal and coworkers mapped the centromeric DNA by chromatin immunoprecipitation approaches of CENP-A (7).

To identify the centromeric DNA sequences of C. auris, Narayanan et al. (7) employed ChIP-seq (chromatin immunoprecipitation followed by next-generation sequencing) of the centromere-specific histone H3 variant Cse4 (also known as CENP-A). C. auris *CSE4* was identified by its homology to C. albicans *CSE4*. A C-terminal protein A tag was then introduced at the endogenous locus of *CSE4* in a representative C. auris clade I strain (B8441). To check the correct localization of Cse4 and the functionality of the tagged version, the authors performed indirect immunofluorescence in mitotic C. auris cells carrying the Cse4-protein A construct. As expected, Cse4-protein A formed one discrete focus per nucleus in nondividing cells and a focus each at the leading edge of dividing daughter nuclei (7); this is indicative of centromere clustering (20). Sequencing of the DNA associated with Cse4-protein A revealed a single peak each on 7 different contigs of the published B8441 genome sequence. The peaks of Cse4-binding (i.e., centromeric) DNA were between ∼2.5 and ∼2.9 kb in size (Fig. 1) and comprised unique DNA sequences which show no homologies to each other or to any sequences within the genome. Centromeric DNAs were found in GC-poor troughs in all instances; the only repeat sequences detected were 40-bp poly(A) and poly(T) stretches in or near all centromeric DNA regions. Genes in the vicinity of centromeres did not show suppressed expression, which according to the authors suggests that pericentromeric heterochromatin is absent in C. auris (7). This situation is thus very similar to C. lusitaniae (see above) (4).

The centromere sizes and positions were corroborated by ChIP-qPCR (ChIP followed by quantitative PCR) in the clade I strain (B8441) harboring the protein A-tagged Cse4 (7). This strategy was then employed to map centromeres in C. auris clade II, III, and IV strains as well as in strains from the other species of the *C. haemulonii* complex to confirm centromere predictions from bioinformatic analyses based on ORF (open reading frame) content, gene synteny, and GC content. Within C. auris, clade II differs most from the other three clades due to major chromosomal rearrangements (7, 18). Interestingly, the related clade II isolates B11220 and CBS10913T contain a large tandem duplication which encompasses the centromeric DNA (7, 18); technically, it is not possible to discern which of those two centromere candidates is the active one. Other clade II strains do not harbor this duplication (18). Intriguingly, centromeric DNA sequences evolve more quickly than intergenic regions as suggested by a high incidence of substitution mutations observed between the different clades (7). The karyotypes of clades I, III, and IV are quite similar, with only 2 major chromosome translocation events detected between clades III and IV. Clade II has undergone multiple chromosome rearrangements, and several of those map to the centromeric regions and break the gene synteny surrounding them (7, 18). This suggests that centromeres can be sources of karyotype diversification in C. auris.

Strains of the other *C. haemulonii* complex species displayed the same centromere properties as C. auris and C. lusitaniae (4, 7). These data together with published genome assemblies allowed the authors to present a convincing hypothesis on chromosome/karyotype evolution of *Clavispora* yeasts. *C. duobushaemulonii* is the only other species with a chromosome-level genome assembly, and its karyotype is very similar to karyotypes of C. auris clades I, III, and IV. This supports the hypothesis that the C. auris clade II karyotype is a derived state, i.e., the C. auris clade likely split off the remaining *C. haemulonii* complex fairly recently. A key difference between C. lusitaniae and the species of the *C. haemulonii* complex is that the former has 8 chromosomes whereas the studied representatives of the latter have 7 chromosomes. Analysis indicated that C. lusitaniae chromosome 8 has been translocated as 3 fragments onto other chromosomes in the last common ancestor of the *C. haemulonii* complex species. The C. lusitaniae centromere 8 can still be detected in the genomes of *C. haemulonii* complex species (Fig. 1) but is inactive in *C. haemulonii* complex species as it has undergone substantial DNA sequence attrition and fails to recruit Cse4 (7). Finally, the authors explore the published genomes of additional species belonging to the *Clavispora* clade, all of which contain 8 potential centromeric regions defined by gene synteny in GC-poor troughs. In Candida heveicola, one of the 8 centromeres showed DNA sequence attrition similar to *C. haemulonii* complex species, whereas the other species studied (Candida blattae, Candida intermedia, Candida oregonensis) had 8 full-length centromeres like C. lusitaniae.

The nonrepetitive nature of centromeric DNA in *Clavispora* raises several intriguing questions about their chromosome biology. It is generally accepted that centromeric DNA repeats are a source of genome instability via ectopic recombination, but it has also been suggested that recombination between centromeric repeats is important for their maintenance (21). When centromeric DNA sequences are unique and nonrepetitive, as in *Clavispora* species, how do they contribute to karyotype diversification? Sanyal and coworkers suggest that due to the proximity of centromeric DNAs in the centromere clusters (7, 20), chromosome rearrangements could be a consequence of replication fork perturbations near centromeres. Maybe the poly(A) and poly(T) stretches play a role as sites for ectopic recombination? In the absence of common DNA sequence signatures, how is centromere identity defined? In C. albicans, centromeres seem to be specified epigenetically as unchromatinized centromeric DNA sequence does not establish a functional centromere (22). But then C. albicans centromeres also display identifying, partially heterochromatic, histone modifications (9); mechanistically imposing such modifications *de novo* presumably enables the formation of neocentromeres (23). Histones at C. lusitaniae centromeres also have specific modifications, albeit none which are typical for heterochromatin (4). Do centromeres of other *Clavispora* yeasts, including C. auris, harbor the same histone modifications as C. lusitaniae? Are these histone modifications indeed defining centromere identity? And finally, if the underlying DNA sequence is not important, why is centromeric DNA attrition observed after chromosome fusions, as is the case with the centromere of C. lusitaniae chromosome 8 in *C. haemulonii* complex species (7)? Answering these questions will not only give us insight into the fascinating chromosome biology of these opportunistic pathogens but will also provide clues to how genetic and genomic diversity is generated to evolve virulence and antimicrobial resistance traits.
